# Cross-species transmission of gastrointestinal parasites between wild and domestic canids

**DOI:** 10.1590/S1984-29612026002

**Published:** 2026-03-16

**Authors:** Ana Camilla Ignácio dos Santos, Antonio Francisco Malheiros, Lucas França de Barros, Daniel Moura Aguiar, Aurea Regina Alves Ignácio, Manoel dos Santos-Filho

**Affiliations:** 1 Universidade do Estado de Mato Grosso – UNEMAT, Faculdade de Ciências Agrárias e Biológicas – FACAB, Programa de Pós-graduação em Ciências Ambientais, Laboratório de Biologia Parasitária – LaBPar, Cáceres, MT, Brasil; 2 Universidade Federal de Mato Grosso – UFMT, Laboratório de Virologia e Rickettsioses, Hospital Veterinário – HOVET, Faculdade de Medicina Veterinária – FAVET, Cuiabá, MT, Brasil; 3 Universidade do Estado de Mato Grosso – UNEMAT, Faculdade de Ciências Agrárias e Biológicas – FACAB, Centro de Pesquisa em Limnologia, Biodiversidade e Etnobiologia do Pantanal – CELBE, Programa de Pós-graduação em Ciências Ambientais, Laboratório de Ecotoxicologia, Cáceres, MT, Brasil; 4 Universidade do Estado de Mato Grosso – UNEMAT, Faculdade de Ciências Agrárias e Biológicas – FACAB, Centro de Pesquisa em Limnologia, Biodiversidade e Etnobiologia do Pantanal – CELBE, Programa de Pós-graduação em Ciências Ambientais, Laboratório de Mastozoologia, Cáceres, MT, Brasil

**Keywords:** Coproparasitological analysis, disease ecology, cross-infection, zoonoses, wildlife conservation, Análise coproparasitológica, ecologia de doenças, infecção cruzada, zoonoses, conservação da vida silvestre

## Abstract

The interface between wild and domestic canids can facilitate cross-transmission of gastrointestinal parasites, with implications for biodiversity conservation and public health. In this study, we evaluated the occurrence and diversity of these parasites in fecal samples from *Cerdocyon thous* (n = 7) collected within the Serra das Araras Ecological Station (EESA) and from domestic dogs (*Canis lupus familiaris*) (n = 10) sampled in surrounding rural properties in the Cerrado biome, Brazil. Four parasite morphotypes were identified in *C. thous* and six in dogs, as well as mite eggs in wild canids’ samples, interpreted as environmental contamination. The most prevalent parasites were *Blastocystis* spp. (57.1%), *Trichuris* spp. (42.8%), *Capillaria* spp. (28.5%), and eggs compatible with Ancylostomatidae (14.2%) in *C. thous*, and *Trichuris* spp. (50%) and *Blastocystis* spp. (40%) in dogs. The overlap between hosts was marked by the presence of *Blastocystis* spp*.*, *Trichuris* spp*.*, and Ancylostomatidae-type eggs in both canids, representing the most relevant evidence of potential cross-species transmission.

## Resumo

A interface entre canídeos silvestres e domésticos pode facilitar a transmissão cruzada de parasitos gastrointestinais, com implicações para a conservação da biodiversidade e para a saúde pública. Neste estudo, avaliamos a ocorrência e a diversidade desses parasitos em amostras fecais de *Cerdocyon thous* (n = 7) coletadas no interior da Estação Ecológica Serra das Araras (EESA) e de cães domésticos (*Canis lupus familiaris*) (n = 10) amostrados em propriedades rurais adjacentes no bioma Cerrado, Brasil. Quatro morfotipos de parasitos foram identificados em *C. thous* e seis em cães, além de ovos de ácaros nas amostras de canídeos silvestres, interpretados como contaminação ambiental. Os parasitos mais prevalentes foram *Blastocystis* spp. (57,1%), *Trichuris* spp. (42,8%), *Capillaria* spp. (28,5%) e ovos compatíveis com Ancylostomatidae (14,2%) em *C. thous*, e *Trichuris* spp. (50%) e *Blastocystis* spp. (40%) em cães. A sobreposição entre os hospedeiros foi marcada pela presença de *Blastocystis* spp., *Trichuris* spp. e ovos do tipo Ancylostomatidae em ambos os canídeos, representando a evidência mais relevante de potencial transmissão entre espécies.

## Introduction

The crab-eating fox (*Cerdocyon thous*) is a widely distributed Neotropical canid found throughout most of Brazil and other South American countries. It typically inhabits open and semi-open environments, such as the Cerrado, Caatinga, Chaco, and ecotonal areas, and is absent from dense forest formations like the continuous Amazon rainforest (Courtenay & Maffei, 2014). Its omnivorous diet, ecological plasticity, and tolerance to anthropogenic landscapes enable its persistence in fragmented and peri-urban areas, where interactions with domestic dogs and humans are increasingly frequent (Lemos et al., 2013). This spatial overlap raises concern over the transmission of gastrointestinal parasites, particularly those with zoonotic potential (Khalifa et al., 2023).

At the domestic–wildlife interface, domestic dogs often act as reservoirs and amplifiers of parasites, whereas wild canids can disperse them across natural habitats. Such interactions increase the likelihood of cross-species transmission and represent a growing conservation concern, as uncontrolled dog movements around protected areas may expose native carnivores to novel parasitic pressures (Corrêa & Passos, 2001; Curi et al., 2017; Thompson et al., 2009). Common protozoa reported in wild and domestic canids include *Blastocystis* spp., *Giardia* spp., and *Sarcocystis* spp., often linked to fecal contamination or complex life cycles involving several developmental stages and, for some taxa, the participation of intermediate hosts (Zorzan et al., 2020; Adam, 2001). Helminths such as *Trichuris* spp., *Capillaria* spp., and Ancylostomatidae may cause anemia, malnutrition, or death, while cestodes like *Dipylidium caninum* are associated with ectoparasite infestations (Khalifa et al., 2023).

Protected areas such as the Serra das Araras Ecological Station (EESA) are key refuges for native carnivores but are increasingly influenced by human activities in surrounding landscapes. Free-ranging dogs may introduce parasites into these environments, affecting wildlife health and population viability, while wild canids may transmit pathogens to domestic hosts. Investigating parasite occurrence and overlap in this context is essential to assess cross-species transmission risks and support integrated conservation and health strategies.

This study aimed to evaluate the diversity and prevalence of gastrointestinal parasites in *C. thous* and domestic dogs within and around EESA. We hypothesised that parasite assemblages would occur in both hosts, indicating potential overlap at the domestic–wildlife interface.

## Material and Methods

### Study area

**Figure 1:**
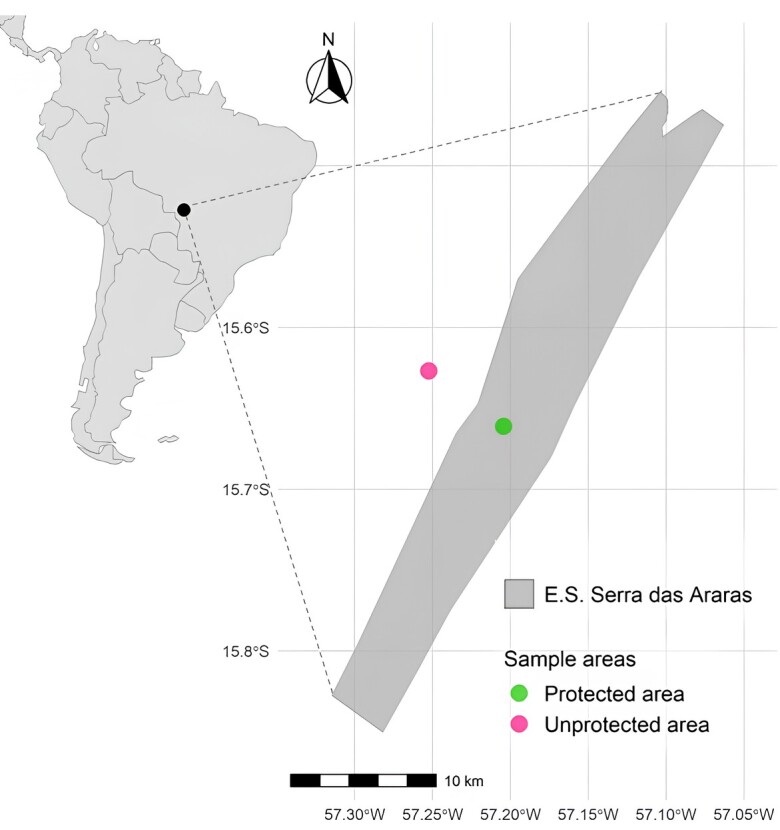
Study site in central Brazil showing the Serra das Araras Ecological Station (EESA) and the adjacent rural community of Salobra Grande.

The study was conducted in the Serra das Araras Ecological Station (EESA), a strictly protected area of 27,159.71 ha in southwestern Mato Grosso, Brazil, and in the adjacent rural community of Salobra Grande (~4 km west of the reserve). EESA is an important refuge for native carnivores and other wildlife within a landscape increasingly modified by agriculture and human occupation. The proximity of rural settlements, where domestic dogs roam freely, facilitfecalates their access to the reserve and contact with wild canids, creating conditions for cross-species parasite transmission. Households sampled for dog feces were located 1–5 km from the EESA boundary, and spatial overlap with wild canids is frequent. The reserve extends between 15°33′–15°39′S and 57°03′–57°19′W, with elevations from 210 to 810 m. Vegetation consists of a Cerrado–forest mosaic (~90%) interspersed with secondary growth, grasslands, and floodplains (Figure 1).

### Capture and sampling procedures

The Serra das Araras Ecological Station (EESA) is bordered by the rural community of Salobra Grande, where domestic dogs (*Canis lupus familiaris*) roam freely and frequently enter the protected area, increasing opportunities for contact with wild canids. Seven crab-eating foxes (*Cerdocyon thous*) were captured within EESA using ten Tomahawk traps (115 × 55 × 60 cm), spaced at least 500 m apart and baited with raw chicken. Traps remained active for three nights during eight sampling sessions. Captured animals were anaesthetised with tiletamine–zolazepam (Zoletil® 50, Virbac; 0.2–0.3 mg/kg IM), weighed, monitored by a veterinarian, and released after recovery. Rectal fecal samples were collected using sterile gloves, ensuring that all wild canid samples originated unequivocally from the captur ed C. thous individuals.

In Salobra Grande, fecal samples were obtained from one free-ranging owned dog per household (n = 10) located within 1–3 km of the EESA boundary. Samples were collected rectally during physical examination to ensure freshness and avoid environmental contamination. All domestic dogs had legal owners, who provided informed consent and assisted during sampling. One dog per household was selected, always an animal living under the same conditions as the others. Fecal samples were obtained by direct digital rectal collection using sterile gloves, without any defecation-inducing methods. Owners manually restrained the animals during the physical examination, allowing safe and unequivocal identification of the sampled individual. All samples were stored in sterile 80 mL tubes labelled by individual ID (*CS* for *C. thous*, *CD* for dogs), refrigerated (2–8 °C), and transported to the laboratory for analysis.

All procedures involving wild and domestic animals were conducted under authorisation for scientific purposes (SISBIO No. 75169-1) and approved by the Animal Ethics Committee (CEUA/UNEMAT, Opinion 006/2020) and Human Research Ethics Committee (CEP/UNEMAT, Opinion 4,355,595).

### Parasitological analysis

Analyses were performed at the LaBPxar laboratory (UNEMAT, Cáceres) using spontaneous sedimentation (Hoffmann et al., 1934) and centrifugal flotation (Faust et al., 1939). In the sedimentation technique, approximately 2 g of feces were emulsified in 7 mL of water, filtered, and left to sediment for 2 h. Slides were examined under a Nikon Eclipse E200 microscope (10× and 40× objectives) at 100× total magnification for initial screening and 400× for detailed identification of parasite eggs, cysts, and oocysts.

For centrifugal flotation, the same tube was centrifuged at 733 × g for 1 min, washed three times, and resuspended in 33% zinc sulfate solution (specific gravity 1.18). The surface film was mounted and examined at 100× and 400× magnification. Protozoan cysts and oocysts (e.g., *Giardia*, *Sarcocystis*) were identified in flotation preparations, while sedimentation served as a complementary method for detecting helminth eggs. Samples were processed within 24 h of collection, stored under refrigeration (~8 °C), and preserved in 70% ethanol until analysis. Parasite identification was based exclusively on morphological characteristics observed under optical microscopy, including egg shape, shell thickness, presence of polar plugs, and internal structures, following the diagnostic criteria of Hoffmann et al. (1934), Faust et al. (1939), and Hendrix & Robinson (2016). Morphometric measurements were not performed.

## Results

Seventeen fecal samples were analysed: ten from domestic dogs and seven from wild *Cerdocyon thous*. Interviews with dog owners indicated that most animals were free-ranging and had routine access to forest edges adjacent to the reserve, although interview data were not analysed quantitatively. Only one dog per household was sampled to ensure data independence.

Among domestic dogs, individual CD05 showed the highest parasite diversity, harbouringsp., thin-shelled eggs compatible with Ancylostomatidae, and *Sarcocystis* sp., whereas CD03 tested negative for all parasites. *Blastocystis* spp. was the most frequent parasite, detected in 60% of dog samples by Hoffmann’s technique and 30% by Faust’s. Other parasites detected in dogs included *Trichuris* spp. (10%), *Dipylidium caninum* (10%), Ancylostomatidae-type eggs (20%), *Giardia* spp. (20%), and *Sarcocystis* spp. (20% by Hoffmann, 10% by Faust).

**Figure 2:**
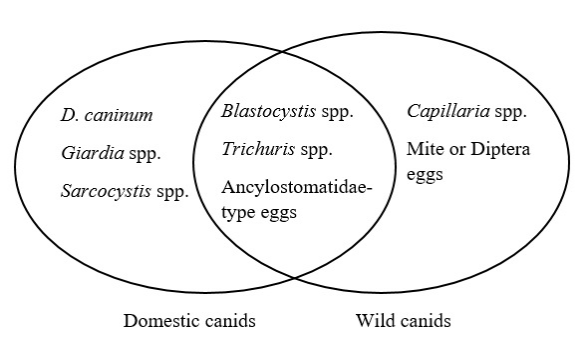
Distribution of exclusive and shared gastrointestinal parasite taxa detected in domestic dogs and *Cerdocyon thous*.

In wild canids, individual CS07 was co-infected with *Blastocystis* sp. and Ancylostomatidae-type eggs, while mite eggs were detected in CS01 and CS03 (Table 1). Ancylostomatidae-type eggs and *Blastocystis* spp. were each detected in 57.1% of *C. thous* samples by Faust’s method, followed by *Trichuris* spp. and *Capillaria* spp. (14.2% each). Mite or Diptera eggs were present in 28.6% of wild individuals and were considered probable environmental contaminants. The synthesis of exclusive and shared parasite taxa between domestic dogs and *Cerdocyon thous* is presented in the Venn diagram (Figure 2), highlighting three parasites common to both hosts (*Blastocystis* spp., *Trichuris* spp., and Ancylostomatidae-type eggs).

**Table 1:** Presence (+) or absence (–) of enteroparasites in domestic (CD) and wild (CS) canids from Salobra Grande community and Serra das Araras Ecological Station (EESA), detected using Hoffman’s (H) and Faust’s (F) coprological techniques.

ANIMAL	*Blastocystis* spp.		*Trichuris* spp		Ancylostomatidae		*Dipylidium caninum*	*Capillaria* spp*.*	*Giardia* spp.		*Sarcocystis* spp.		Mite eggs
	F	H	F	H	F	H	H	F	F	H	F	H	H
CD 01	**-**	**+**	**-**	**+**	**-**	**-**	**-**	**-**	**-**	**-**	**-**	**-**	**-**
CD 02	**-**	**-**	**-**	**-**	**-**	**+**	**+**	**-**	**-**	**-**	**-**	**-**	**-**
CD 03	**-**	**-**	**-**	**-**	**-**	**-**	**-**	**-**	**-**	**-**	**-**	**-**	**-**
CD 04	**+**	**-**	**-**	**-**	**-**	**-**	**-**	**-**	**-**	**+**	**-**	**-**	**-**
CD 05	**-**	**+**	**-**	**-**	**-**	**+**	**-**	**-**	**-**	**-**	**-**	**+**	**-**
CD 06	**-**	**+**	**-**	**-**	**-**	**-**	**-**	**-**	**-**	**-**	**-**	**-**	**-**
CD 07	**+**	**+**	**-**	**-**	**-**	**-**	**-**	**-**	**-**	**-**	**-**	**+**	**-**
CD 08	**-**	**+**	**-**	**-**	**-**	**-**	**-**	**-**	**-**	**+**	**+**	**-**	**-**
CD 09	**-**	**-**	**-**	**-**	**-**	**-**	**-**	**-**	**-**	**-**	**-**	**-**	**-**
CD 10	**+**	**+**	**-**	**-**	**-**	**-**	**-**	**-**	**-**	**-**	**-**	**-**	**-**
CS 01	**-**	**-**	**-**	**-**	**-**	**-**	**-**	**+**	**-**	**-**	**-**	**-**	**+**
CS 02	**+**	**+**	**-**	**-**	**+**	**+**	**-**	**-**	**-**	**-**	**-**	**-**	**-**
CS 03	**-**	**-**	**-**	**-**	**+**	**+**	**-**	**-**	**-**	**-**	**-**	**-**	**+**
CS 04	**+**	**-**	**+**	**+**	**-**	**-**	**-**	**-**	**-**	**-**	**-**	**-**	**-**
CS 05	**+**	**-**	**-**	**-**	**-**	**-**	**-**	**-**	**-**	**-**	**-**	**-**	**-**
CS 06	**-**	**-**	**-**	**-**	**-**	**-**	**-**	**-**	**-**	**-**	**-**	**-**	**-**
CS 07	**+**	**+**	**-**	**-**	**+**	**+**	**-**	**-**	**-**	**-**	**-**	**-**	**-**

Full results by technique are summarised in Table 1. Reports were provided to dog owners/guardians and to the EESA management team.

## Discussion

The close proximity and interaction between domestic and wild animals represent one of the main contemporary threats to wildlife health, facilitating the circulation of parasites and other infectious agents across ecological boundaries (Corrêa & Passos, 2001). Domestic dogs, in particular, are well-known reservoirs and amplifiers of pathogens, and their unrestricted movement into natural habitats promotes parasite exchange with wildlife populations (Santos & Castro, 2006; Curi et al., 2017). Our results indicate that gastrointestinal parasites may be shared between domestic dogs and *Cerdocyon thous* in and around a Cerrado protected area, highlighting the permeability of this interface and its potential implications for conservation and public health.

*Blastocystis* spp. was the most frequently detected protozoan in both host groups. Although traditionally considered a pathogen, growing evidence suggests that it is predominantly a commensal component of the intestinal microbiota and that symptomatic infections are uncommon (Duda et al., 1998; Gill et al., 2013). Its frequent detection in humans and domestic animals highlights its ubiquity in anthropized environments and its utility as an indicator of fecal contamination (Hublin et al., 2021). The relatively high prevalence observed here (~60% in domestic dogs and 57.1% in wild canids) suggests significant environmental contamination and points to the role of spatial overlap in shaping transmission dynamics. Detection in *C. thous* likely reflects exposure to contaminated soil or water and suggests that parasite flow may occur even within strictly protected areas. Comparable prevalence has been reported in free-ranging dogs and wild foxes elsewhere in South America (Hublin et al., 2021) , supporting the hypothesis that *Blastocystis* transmission is facilitated by shared environmental resources.

Thin-shelled eggs compatible with the family Ancylostomatidae were also common, particularly in *C. thous*. Hookworm eggs are morphologically indistinguishable, so our results are limited to family-level identification. Nevertheless, their presence suggests sustained exposure to infective larvae in shared environments, most likely linked to soil contamination by dog feces (Traversa, 2012; Macpherson, 2013). Similar findings have been reported in other regions of Brazil, where free-ranging dogs near forest fragments and conservation units exhibit high hookworm prevalence (Curi et al., 2017; Santos et al., 2012). These results highlight the potential role of domestic dogs as reservoirs of generalist parasites capable of persisting in protected landscapes. The higher occurrence in wild canids may reflect cumulative exposure due to their larger home ranges and continuous contact with contaminated substrates (Thompson et al., 2010).

The detection of *Trichuris* spp. in both hosts reinforces the importance of shared environments in sustaining transmission. The eggs of this nematode are highly resistant and can remain viable in the soil for extended periods, enabling transmission even in the absence of direct contact between hosts (Traversa, 2012). Similarly, the presence of *Dipylidium caninum* indicates that intermediate hosts, such as fleas, likely mediate parasite circulation in both domestic and wild canids (Macpherson, 2013). These findings underscore the complexity of parasite assemblages circulating at the domestic–wildlife interface and the multiple ecological mechanisms that sustain them.

The detection of mite or Diptera eggs in *C. thous* was likely incidental and not indicative of active parasitism. However, it highlights the importance of interpreting parasitological data cautiously, as accidental ingestion or environmental contamination can result in misleading host–parasite associations (Thompson et al., 2009). Furthermore, the inability to differentiate some taxa morphologically, such as Ancylostomatidae eggs, underscores the need for molecular tools to resolve species identity and refine inferences about transmission pathways. Future research integrating molecular diagnostics will be critical to confirm whether the same parasite lineages circulate among domestic and wild hosts.

Our results align with broader patterns reported in Neotropical ecosystems, where spatial overlap, environmental contamination, and behavioural interactions facilitate parasite exchange between domestic and wild carnivores (Macpherson, 2013; Curi et al., 2017). The co-occurrence of shared parasite taxa provides strong evidence of potential cross-species transmission and demonstrates that parasite exchange is an ongoing ecological process shaped by habitat use, host behaviour, and the persistence of infective stages in the environment. These dynamics pose significant conservation challenges, as parasite flow between domestic and wild hosts can reduce population fitness, alter trophic and behavioural interactions, and compromise the long-term viability of native species (Clark et al., 2013; Thompson et al., 2010).

From a management perspective, these findings emphasise the need for integrated strategies to mitigate parasite transmission at the domestic–wildlife interface. Effective measures include regular deworming and vaccination of domestic dogs, active monitoring of free-ranging populations, and public awareness campaigns to promote responsible ownership (Gortázar et al., 2007). In addition, restricting the access of domestic dogs to Conservation Units, combined with owner sensitisation and compliance with local regulations, may help reduce the circulation of parasites at the domestic–wildlife interface. These actions are consistent with the One Health framework, which recognises the interconnectedness of human, animal, and ecosystem health (Zinsstag et al., 2011).

The risks identified here extend beyond *C. thous*, potentially affecting other wild canids of conservation concern in the Cerrado, such as *Speothos venaticus*, *Chrysocyon brachyurus*, and *Lycalopex vetulus*, all of which are susceptible to similar parasites. Strengthening sanitary surveillance and implementing coordinated management actions are essential to prevent pathogen spillover and safeguard vulnerable carnivore populations. Future studies should integrate parasitology, spatial ecology, and molecular tools to deepen our understanding of tr ansmission dynamics and inform evidence-based conservation policies.

## Conclusions

The occurrence of parasitic taxa shared between domestic and wild canids highlights the potential for cross-species transmission at the domestic–wildlife interface. While wild canids showed higher parasite diversity, the occurrence of shared taxa highlights the potential for cross-species transmission at the domestic–wildlife interface. However, because eggs, cysts, and oocysts are often morphologically similar across different parasite species, some identifications remain limited to the family or genus level, and the observed overlap should be interpreted cautiously. These findings reinforce the need for sanitary management and monitoring in protected areas under increasing anthropogenic pressure.

## References

[B001] Adam RD (2001). Biology of *Giardia lamblia.*. Clin Microbiol Rev.

[B002] Clark CG, van der Giezen M, Alfellani MA, Stensvold CR (2013). Recent developments in *Blastocystis* research. Adv Parasitol.

[B003] Corrêa SHR, Passos EC, Fowler ME, Cubas ZS (2001). Biology, medicine, and surgery of South American wild animals..

[B004] Courtenay O, Maffei L (2014). Cerdocyon thous. The IUCN Red List of Threatened Species. Version 2014.3.

[B005] Curi NHA, Paschoal AMO, Massara RL, Santos HA, Guimarães MP, Passamani M (2017). Risk factors for gastrointestinal parasite infections of dogs living around protected areas of the Atlantic Forest: implications for human and wildlife health. Braz J Biol.

[B006] Duda A, Stenzel PFL, Boreham PF (1998). Detection of *Blastocystis* sp. in domestic dogs and cats. Vet Parasitol.

[B007] Faust EC, Sawitz W, Tobie J, Odom V, Peres C, Lincicome DR (1939). Comparative efficiency of various techniques for the diagnosis of protozoa and helminths in feces. J Parasitol.

[B008] Gill FF, Barros MJ, Macedo NA, Junior CG, Redoan R, Busatti H (2013). Prevalence of intestinal parasitism and associated symptomatology among hemodialysis patients. Rev Inst Med Trop São Paulo.

[B009] Gortázar C, Ferroglio E, Höfle U, Frölich K, Vicente J (2007). Diseases shared between wildlife and livestock: a European perspective. Eur J Wildl Res.

[B010] Hendrix CM, Robinson E (2016). Diagnostic parasitology for veterinary technicians..

[B011] Hoffmann WA, Pons JA, Janer JL (1934). Sedimentation–concentration method in *Schistosomiasis mansoni.*. J Public Health.

[B012] Hublin JSY, Maloney JG, Santin M (2021). *Blastocystis* in domesticated and wild mammals and birds. Res Vet Sci.

[B013] Khalifa MM, Fouad EA, Kamel NO, Auda HM, El-Bahy MM, Ramadan RM (2023). Dogs as a source for the spreading of enteric parasites including zoonotic ones in Giza Province, Egypt. Res Vet Sci.

[B014] Lemos FG, Azevedo FCC, Beisiegel BM, Paula RC, Rodrigues FHG, Jorge RSP (2013). Avaliação do risco de extinção do cachorro-do-mato *Cerdocyon thous* (Linnaeus, 1766) no Brasil. Biodivers Bras.

[B015] Macpherson CNL (2013). The epidemiology and public health importance of toxocariasis: a zoonosis of global importance. Int J Parasitol.

[B016] Santos JLC, Magalhães NB, Santos HA, Ribeiro RR, Guimarães MP (2012). Parasites of domestic and wild canids in the region of Serra do Cipó National Park, Brazil. Rev Bras Parasitol Vet.

[B017] Santos SV, Castro JM (2006). Ocorrência de agentes parasitários com potencial zoonótico de transmissão em fezes de cães domiciliados do município de Guarulhos, SP. Arq Inst Biol.

[B018] Thompson RCA, Kutz SJ, Smith A (2009). Parasite zoonoses and wildlife: emerging issues. Int J Environ Res Public Health.

[B019] Thompson RCA, Kutz SJ, Smith A (2010). Parasite zoonoses and wildlife: emerging issues. Int J Parasitol.

[B020] Traversa D (2012). Pet roundworms and hookworms: a continuing need for global worming. Parasit Vectors.

[B021] Zinsstag J, Schelling E, Waltner-Toews D, Tanner M (2011). From “one medicine” to “One Health” and systemic approaches to health and well-being. Prev Vet Med.

[B022] Zorzan WNM, Moreira LM, Silva SL, Ikeda SKC, Lima MF, Silva MEV (2020). Prevalência de *Blastocystis* spp. provenientes de amostras fecais de moradores de dois biomas mato-grossenses. Gaia Sci.

